# Molecular Identification and Characterization of *Fusarium* Associated with Walnut Branch Blight Disease in China

**DOI:** 10.3390/pathogens12070970

**Published:** 2023-07-24

**Authors:** Ting Ma, Chengde Yang, Fengfeng Cai, Richard Osei

**Affiliations:** Biocontrol Engineering Laboratory of Crop Diseases and Pests, College of Plant Protection, Gansu Agricultural University, Lanzhou 730070, China; mating251525@gmail.com (T.M.); 18394280709@163.com (F.C.); nanasei2000@gmail.com (R.O.)

**Keywords:** walnut branch blight, morphological, molecular, systematics, disease control

## Abstract

In October 2020, samples of walnut branch blight were collected from Longnan. Pathogens were isolated and identified based on morphological and molecular features, and their characteristics were analyzed by pathogenicity. Pathogenicity testing revealed that seven strains (LN-1, LN-3, LN-6, LN-19, LN-27, QY3-1, and QY9-1) induced symptoms of walnut branch blight that were consistent with those observed in the field after inoculation. Furthermore, some *Fusarium*-type conidia and spherical chlamydospores were visible indicating that they were *Fusarium* spp. A molecular characterization including sequence and phylogenetic analysis of the *ITS*, *TEF-1α*, *βTUB*, *Fu,* and *LSU* gene regions revealed that LN-1 and LN-19 belonged to *F. avenaceum*, LN-3 and LN-6 to *F. acuminatum*, LN-27 to *F. sporotrichioides*, and QY3-1 and QY9-1 to *F. tricinctum*. This is the first time that *F. acuminatum*-, *F. sporotrichioides-*, and *F. tricinctum*-caused walnut branch blight has been reported in China.

## 1. Introduction

The walnut (*Juglans regia* L.) is a valuable economic tree due to its nuts and wood. The walnut kernel, a rich source of several beneficial chemicals, contributes to more than 40% of the weight of the nuts [1,2]. Walnut plantations can provide significant economic and environmental benefits [3]. The walnut is presently grown commercially in Southern Europe, Northern Africa, Eastern Asia, the United States, Western South America, and China. China is the world’s greatest producer of walnuts [4], accounting for approximately 48% of global production [5]. Walnut, on the other hand, is commonly affected with a variety of diseases such as cankers, blights, anthracnose, dieback, and kernel decay [6,7,8,9,10,11,12,13,14,15,16]. Walnut branch blight (disease incidence of 3%) is widely documented to be a rising concern for walnut farmers in Gansu, Xinjiang, Henan, and other Chinese provinces, hurting fruit quality and output [6]. Walnut branch blight offers a possible danger to the further development of the walnut industry in China, although there is little precise information regarding the incidence of walnut branch blight and the pathogen diversity in China.

At least 10 species of pathogens are known to cause diseases in walnut worldwide, including *Colletotrichum godetiae* [7], Botryosphaeriaceae and Diaporthaceae species [8,9,10], *Cytospora atrocirrhata*, *Colletotrichum chrysosperma*, *Colletotrichum gigalocus*, *Colletotrichum nivea*, *Colletotrichum sacculus* [11,12], *Boeremia exigua* [13], *Fusarium solani* [14], *Fusarium incarnatum* [15], and *Fusarium semitectum* [16]. However, *Fusarium* is rarely reported on walnut branch blight. *Fusarium*, a soilborne fungal genus with a large host range and significant economic relevance as a plant pathogen, comprises a large number of morphologically and phylogenetically different, widely distributed fungal species [17,18]. Traditionally, *Fusarium* species were recognized based on colony morphology, conidial morphology such as macro- and microconidia size and shape, and other phenotypic characteristics such as the presence and placement of chlamydospores (intercalary or terminal) [17,19]. *Fusarium* species established using phylogenetic procedures are also exceedingly difficult to identify using solely traditional morphological criteria. For detecting and discriminating closely related *Fusarium* species, molecular-based techniques have proven particularly effective [20]. Sequences from the nuclear ribosomal DNA’s internal transcribed spacer *(ITS*) region are commonly employed in phylogenetic investigations of numerous fungi. The *ITS* region has not been commonly employed in phylogenetic analyses of *Fusarium* and allied fungi since the revelation that species in the *Fusarium fujikuroi* and *Fusarium oxysporum* species complexes contain non-orthologous copies of the ITS2, which can lead to inaccurate phylogenetic results [21]. The β-tubulin (*βTUB*) [21,22] and translation elongation factor 1-alpha (*TEF1-α*) nuclear genes [23,24] have been effectively employed to differentiate a variety of species within the *Fusarium* genus.

Studies on etiology are essential for a good disease diagnosis to develop future research on the epidemiology and control of the disease. Therefore, this study used a polyphasic strategy that incorporated morphological features and multi-gene phylogenetic analysis to identify pathogens causing walnut branch blight in China.

## 2. Materials and Methods

### 2.1. Isolation of Fungal Cultures

**Figure 1:**
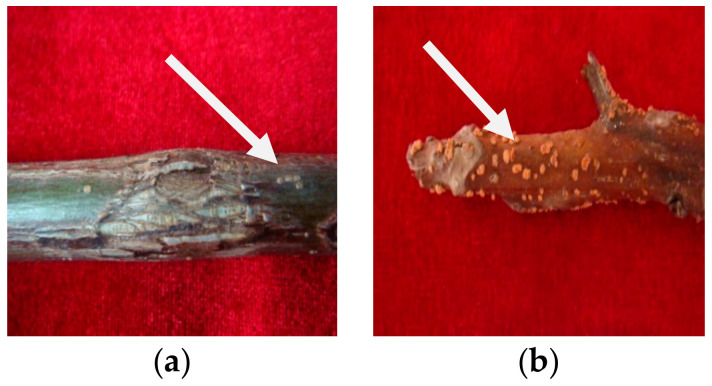
Epidermal symptoms of walnut branch blight: (**a**) withered and irregularly cracked, (**b**) dense yellow dots.

Walnut branch blight, which develops in 1–2-year-old shoots, was identified in 2020 in Longnan City, Gansu Province. Symptoms of the disease were fusiform or oval black lesions that gradually developed and extended on the branches, blight and dieback of branches, reddish-brown dead branch bark with plenty of yellow tiny dots (sporodochium), and defoliation. In October 2020, 30 branches with typical symptoms (Figure 1) were collected from the LinYuan family farm walnut orchard in Longnan City, Gansu Province, China. A stereomicroscope was used to investigate the fungal structures on the lesions with the bark layer.

To isolate the pathogen, walnut branches were washed with tap water and dried. Branch segments (3 × 3 mm) were removed and surface-sterilized for 45 s with 75% ethanol, encompassing the margin of a lesion and nearby asymptomatic tissues. After three washes with sterile distilled water, samples were plated onto potato dextrose agar (PDA) and cultured at 25 °C for 5 days.

### 2.2. Morphological Identification

Single hyphal tips from *Fusarium* species colonies with typical growth characteristics were transferred to fresh PDA and cultured to obtain pure cultures. The strains were cultured for 5 days at 25 °C on PDA, and the texture, border, color, and other properties of the colonies were noted. After 15 days, the size of the conidia was measured. Using a No.333 light microscope (Carl Zeiss, Suzhou, China), 50 conidia and 50 chlamydospores were photographed and measured. Structures were measured using light microscopy with 40× and 100× objective lenses.

### 2.3. Pathogenicity Tests

The representative strains from all identified *Fusarium* species were tested for pathogenicity on walnut branches (approximately 15 cm long and 1 cm in diameter). First, in vitro pathogenicity tests were performed on healthy 1-year-old branches of walnut “QingXiang” in the field. Walnut branches were surface-disinfected with 70% ethanol before being punctured gently with a sterilized insect needle (No. 5) to generate a wound (about 1 mm). The strains were planted on PDA plates and cultured for 5 days at 25 °C before spraying 300 μL of their spore suspensions (1.0 × 10^6^ CFU/mL) over the wounded plant tissue [25]. As a negative control, sterile water was used. To retain moisture, all treated branches were placed in transparent plastic boxes with lids containing wet sterile filter paper. The same method described above was used to perform in vivo pathogenicity tests on 2-year-old tree-detached branches. They were also kept in an artificial climate chamber (25 ± 1 °C and humidity 100%). The symptoms were examined and photographed using a digital camera (SONY DSC-T20, Tokyo, Japan) regularly. To confirm Koch’s postulates, the strains were re-isolated from inoculated branches and pathogenicity tests were performed on healthy branches. Each test was performed in triplicate.

### 2.4. DNA Extraction, Polymerase Chain Reaction Amplification, and DNA Sequencing

Using the E.Z.N.A.^TM^ HP Fungal DNA Kit (Omega, Shanghai, China), genomic DNA was extracted from strains of hyphae scraped from PDA cultures (5 days old) and stored at −20 °C.

The internal transcribed spacer (*ITS*), translation elongation factor 1-α (*TEF-1α*), β-tubulin (*βTUB*), *Fu* (a pair of genus-specific primer Fu3/Fu4 for *Fusarium* was designed, based on 18S rDNA and internal transcribed spacer ITS2 sequence), and large subunit rDNA (*LSU*) genes were amplified using ITS1/ITS4 primers [26], TEF1αF/TEF1αR primers [20], btαa/btαb primers [27], Fu3/Fu4 [28], and LR0R/LR5 [29], respectively. PCR was performed on a thermal cycler (Model A37028; Shanghai, China) in a 25 μL volume including 1 μL of DNA template, 1 μL of each primer, 12.5 μL of 2× Easy TaqPCR Super Mix, and 9.5 μL of ddH_2_O (Qingke Biotech, Xi’an, China). However, five different genes were amplified under different conditions. The amplified condition for the *ITS* was 30 s initial denaturation at 95 °C, followed by 30 cycles of 98 °C for 10 s, 58 °C for 30 s, 72 °C for 1 min, and a final elongation step of 2 min at 72 °C; and the condition for the other genes were similar to that of the *ITS* except for annealing at 55 °C for 30 s and cycling of 35 times of the *TEF-1α*, annealing at 58 °C for 30 s and cycling of 34 times of the *βTUB*, annealing at 55 °C for 30 s and cycling of 33 times of the *Fu*, and annealing at 48 °C for 30 s and cycling of 35 times of the *LSU*, respectively. Qingke Biotech (Shaanxi, China) visualised the PCR amplification results on 1% agarose gels and sequenced the amplicons three times. The sequences were entered into the GenBank database.

### 2.5. Phylogenetic Analysis

The sequences were BLASTed against Genbank database by the National Center for Biotechnology Information (NCBI: http://www.ncbi.nlm.nih.gov; Accessed on 20 June 2021) nucleotide database. The gene sequences were aligned with homologous sequences from GenBank using the multiple sequence alignment programme (ClustalX 2.0). Ambiguously aligned characters and gaps were removed using default settings. Sequences of closely related *Fusarium* spp. strains were obtained from GenBank to construct datasets for phylogenetic analysis.

GenBank sequences were added to the sequences obtained, and a phylogenetic tree was constructed with multi-gene phylogenetic analysis using MEGA11.0 and the maximum likelihood analysis with 1000 bootstrap replications. Values greater than 50% are displayed.

## 3. Results

### 3.1. Isolation of Fungi

A total of 30 fungal colonies were isolated from the infected branches based on the colony characteristics that formed on PDA plates 5 days after inoculation. These included eight strains of *Boeremia* spp., six strains of *Alternaria alternata*, fourteen strains of *Fusarium,* and two strains of *Melanconium oblongum*, and the isolation frequency was 26.7%, 20%, 46.7%, and 6.7% each, respectively. The morphological and cultural characteristics of the strains on PDA plates, with curved conidia and single chlamydospores, which were similar to the morphology of strains isolated directly from typical symptoms of branch blight, led to the initial identification of seven fungal strains as *Fusarium* spp. in this current study. As a result, the strains were given the names LN-1, LN-3, LN-6, LN-19, LN-27, QY3-1, and QY9-1, and were used for further study.

This is a preliminary study and further investigation is required to determine if these *Fusarium* species are found in other parts of the Gansu province.

### 3.2. Morphological and Cultural Characteristics

**Figure 2:**
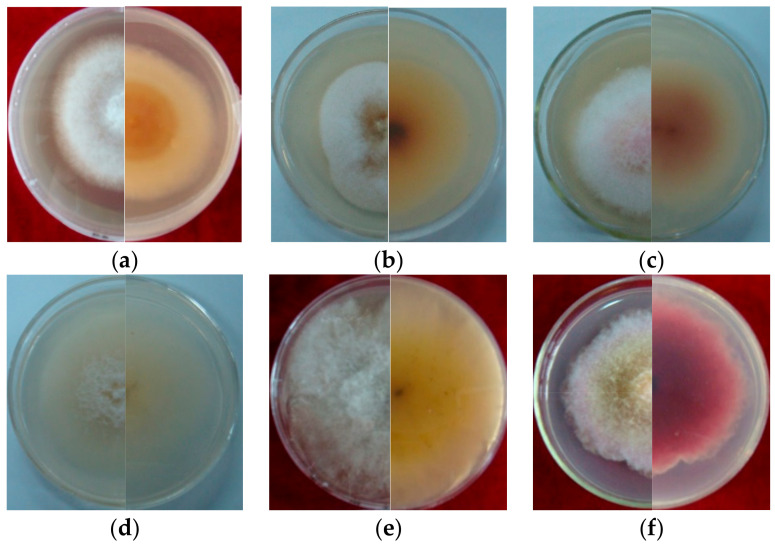

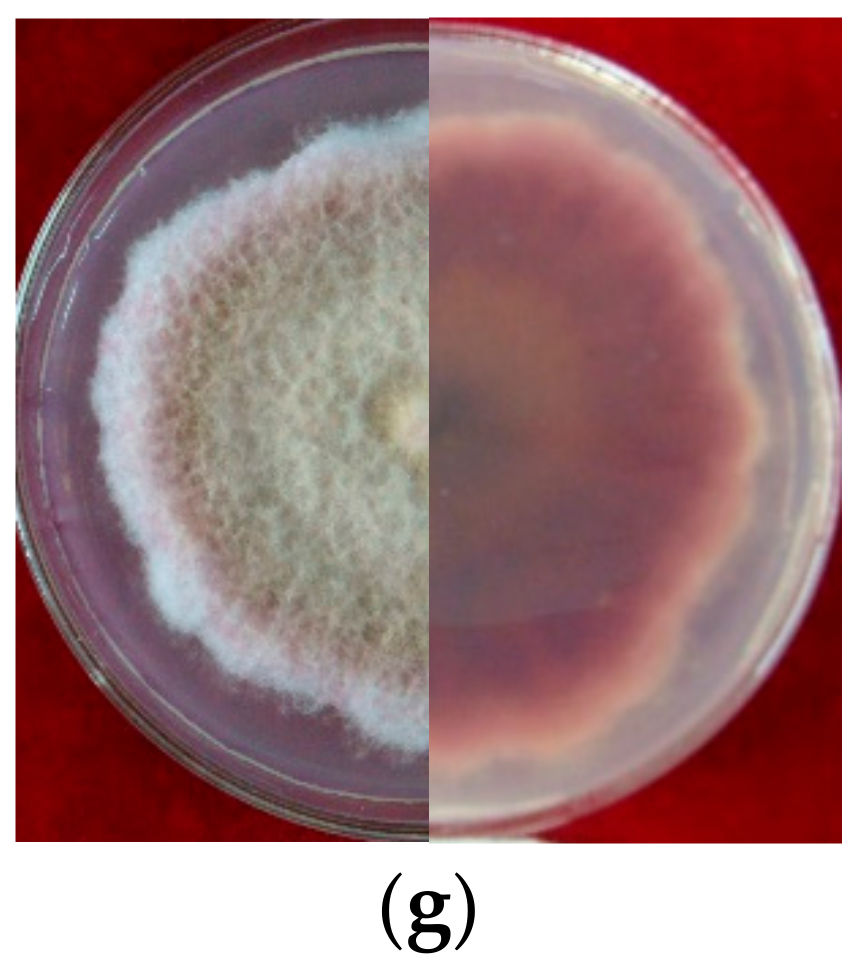
Cultural characteristic of *Fusarium* spp. growth on PDA plate for 5 days (left, upper view; right, dorsal view): (**a**): LN-1; (**b**): LN-19; (**c**): LN-3; (**d**): LN-6; (**e**): LN-27; (**f**): QY3-1; (**g**): QY9-1.

**Figure 3:**
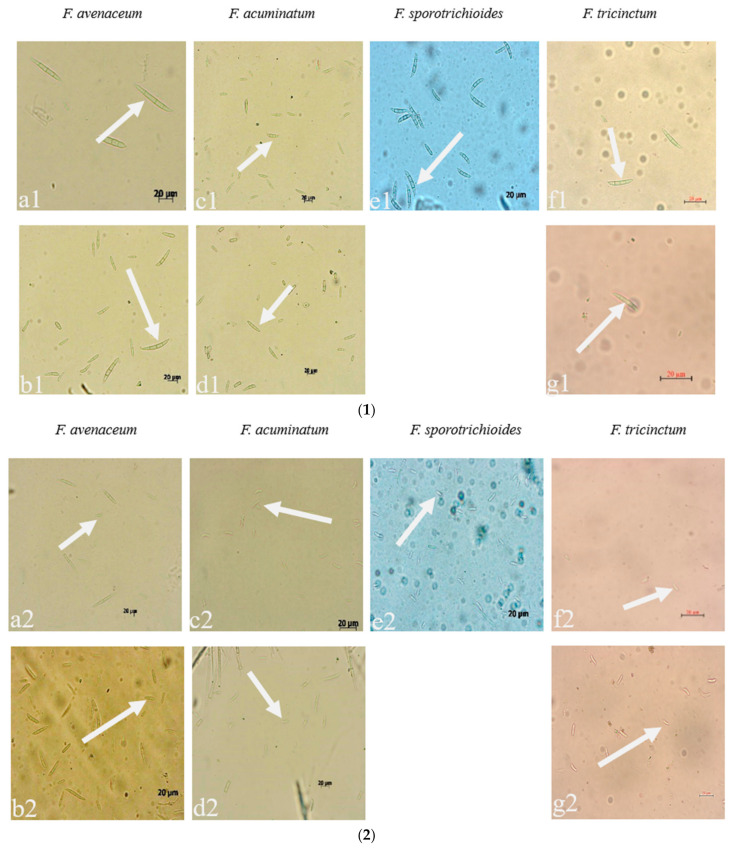

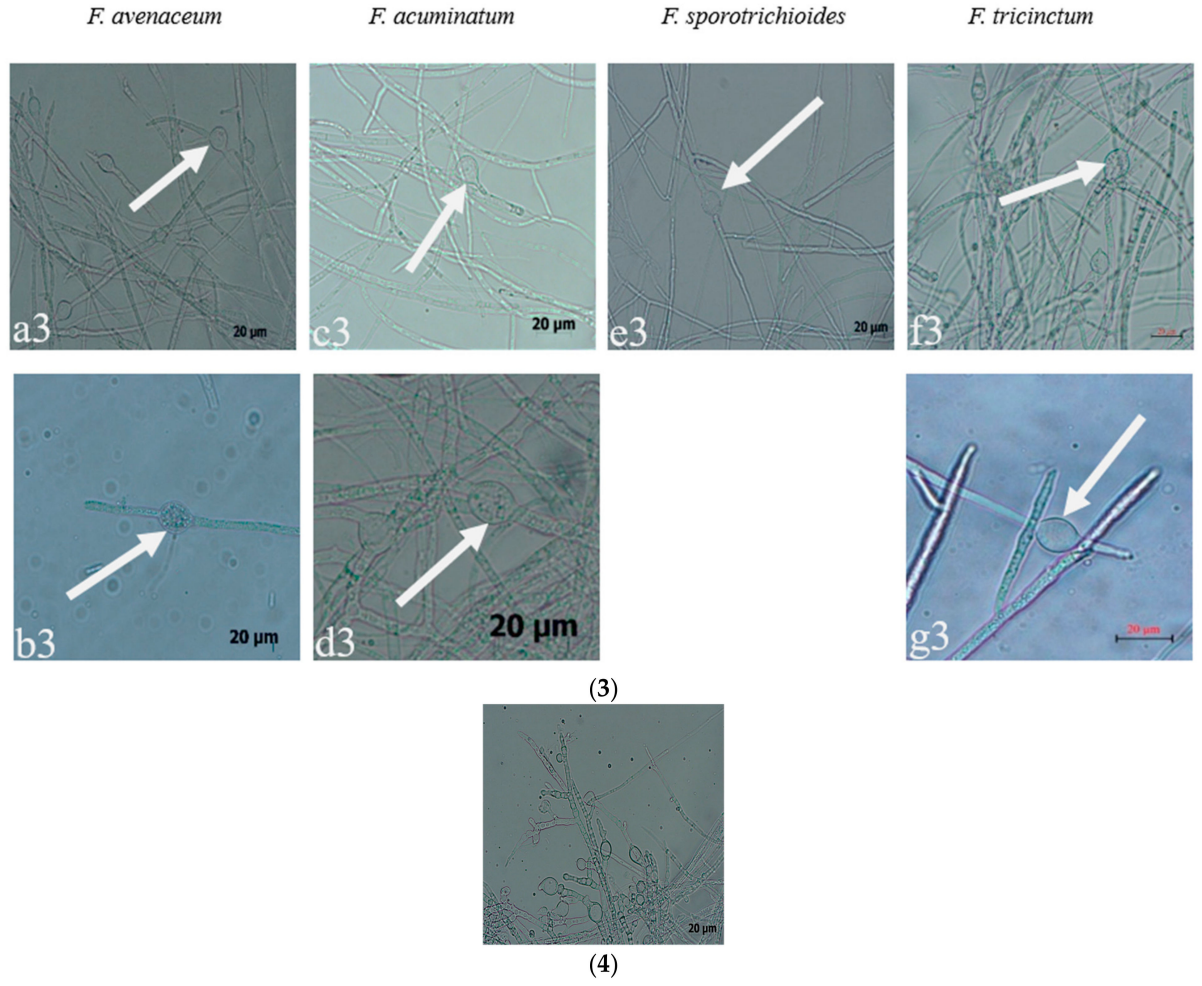
The conidia, chlamydospores, and conidiogenous cell of *Fusarium* species: (**1**) macroconidia; (**2**) microconidia; (**3**) chlamydospores; (**4**) conidiogenous cell; (**a**–**g**): the pathogens LN-1, LN-19, LN-3, LN-6, LN-27, QY3-1, and QY9-1.

Seven strains are maintained as culture collections in the laboratory of Plant Pathogenic Bacteria and Bacterial Diversity, College of Plant Protection, Gansu Agricultural University. On PDA, fungal colonies grew up to 80 mm in diameter within 7 days at 25°C. LN-1 and LN-19 developed rich white colonies that were densely packed with hyphae. LN-3 and LN-6 colonies grew quickly and changed from white to orange. LN-27 grew quickly, with white flocculence that finally became yellow. QY3-1 and QY9-1 have yellow-brown centers and pink-to-white edges, with dark red pigments in the agar (Figure 2). After 15 d of incubation on PDA at 25 °C in darkness, conidia were seen under a microscope. Tested strains produced three types of spores: macroconidia (Figure 3a), microconidia (Figure 3b), and chlamydospores (Figure 3c). The macroconidia were sickle-shaped, with three to five septa, the majority of which had three septa, whereas the microconidia were ellipsoid to fusoid, with zero to one septum, the majority of which had one septum (Table 1). The chlamydospores appeared to be spherical, intercalary, and formed in chains with a smooth exine in the cultures. Conidiogenous cells are hyaline and bottle-shaped, with more polyphialide open ends from which conidia are generated while the length of the phialide remains constant (Figure 3d). Based on these morphological characteristics, the strains were identified as *Fusarium* spp.

Based on morphological similarities with previous descriptions of the colony, conidiogenous cell, conidia, and chlamydospores [30], the strains were provisionally classified as *Fusarium* spp.

### 3.3. Pathogenicity Tests

**Figure 4:**
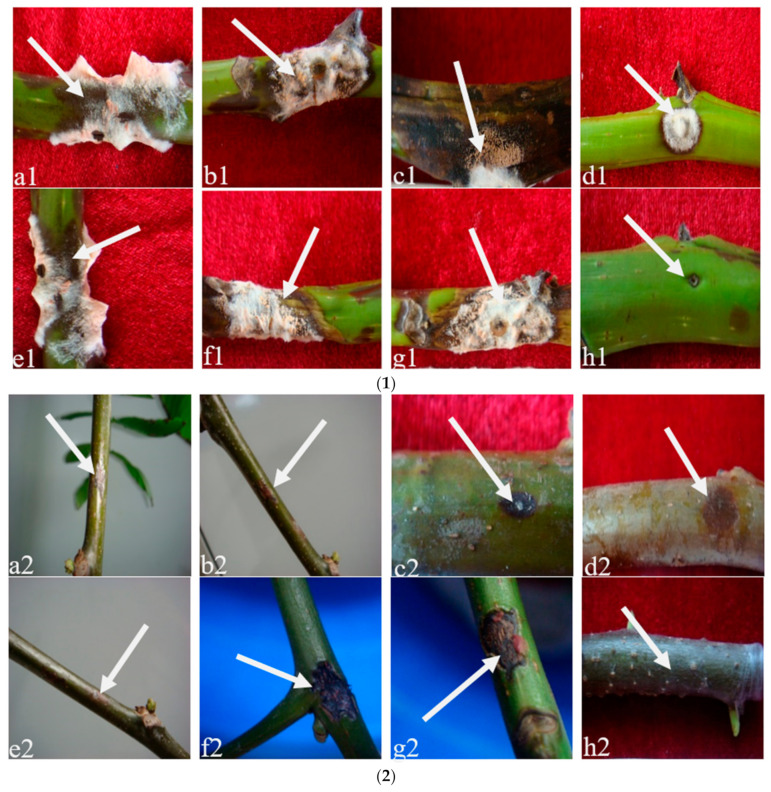
Pathogenicity test of *Fusarium* species: (**1**) in vitro (branch segment on agar); (**2**) in planta (branch attached to tree); (**a1**–**h1**): the vitro pathogenicity of the pathogen LN-1, LN-19, LN-3, LN-6, LN-27, QY3-1, QY9-1, and CK; (**a2**–**h2**): the planta pathogenicity of the pathogen LN-1, LN-19, LN-3, LN-6, LN-27, QY3-1, QY9-1, and CK.

All strains tested were able to induce lesions on the inoculated walnut branches after inoculation in laboratory conditions (Figure 4). The inoculated plants developed branch blight symptoms 3 days later, with dark brown to black and depressed lesions 5 days later. As expected, the symptoms around the wound progressed from dark gray to reddish-brown, with some yellow spots forming on the uneven lesions. The expressed symptoms were typical for walnut branch blight, as described. Non-inoculated branches remained symptomless under identical conditions. The fungus successfully re-isolated from diseased walnut branch tissues and was reidentified as *Fusarium* spp. based on its morphological and molecular characteristics, confirming Koch’s postulates (Figure S1). The results showed that the seven strains caused the symptoms of walnut branch blight.

### 3.4. Molecular Identification

**Figure 5:**
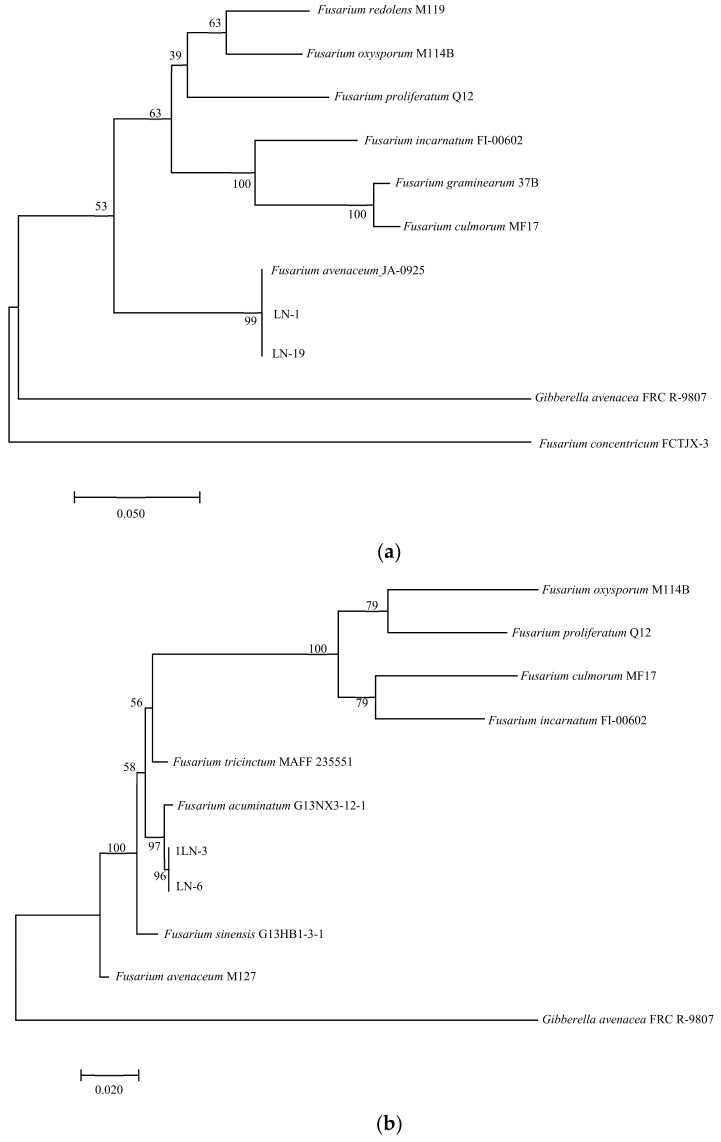

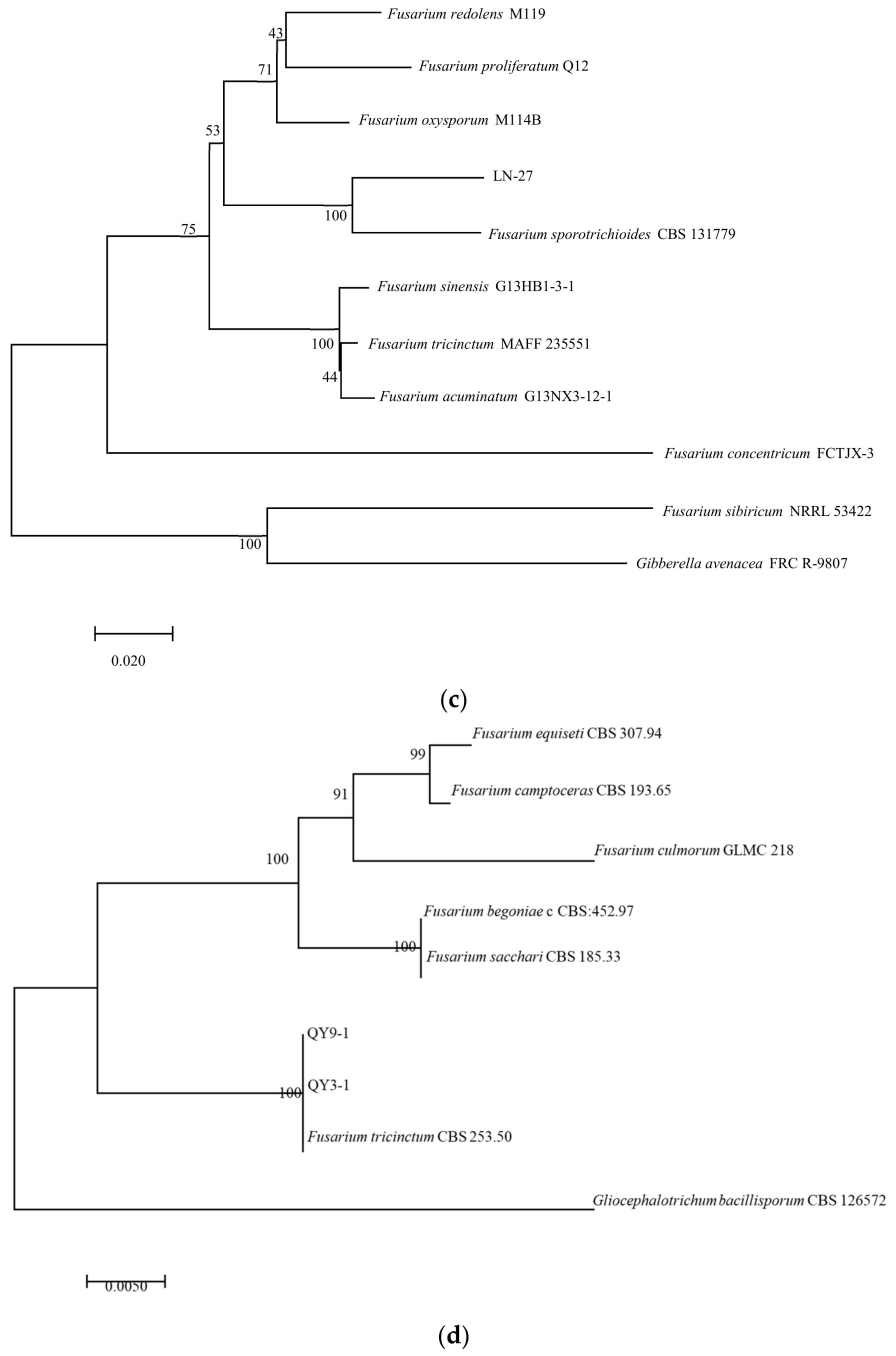
Maximum likelihood (ML) tree of *Fusarium* based on a combined five-gene data set (*ITS, TEF-1α, βTUB, Fu, and LSU*) of the MEGA 11.0 program. Values above nodes are bootstrap values obtained from 1000 replicates. Values greater than 50% are displayed. (**a**) *F. avenaceum*; (**b**): *F. acuminatum*; (**c**): *F. sporotrichioides*; (**d**): *F. tricinctum*. Combining obtained results of morphological identification and molecular characterization, it can be concluded that all strains belong to *F. avenaceum*, *F. acuminatum*, *F. sporotrichioides,* and *F. tricinctum*, respectively.

In PCR analysis, fragments of 530, 680, 300, 570, and 900 bp from *ITS*, *TEF-1α*, *βTUB*, *Fu*, and *LSU* genes were amplified in all tested strains. To identify fungal species in these strains, PCR products were sequenced, and the sequences were then deposited in the NCBI database (Table 2). The *ITS*, *TEF-1α,* and *βTUB* sequences of LN-1 (GenBank accession numbers: MT239572, MT276173, and MT276177) and LN-19 (GenBank accession numbers: MT239575, MT276176, and MT276180) shared a 100%, 99.7%, and 98.73%, and 100%, 99.71%, and 99.68% identity with the corresponding sequences of *F. avenaceum* (GenBank accession numbers: MN522841, MG670380, and KU852602, and MN186746, MK111429, and KP170733), respectively. A multilocus phylogenetic tree of *F. avenaceum* was constructed, and the results revealed that the grouping of strains LN-1 and LN-19 were supported by a 100% bootstrap value (Figure 5a). The *ITS*, *TEF-1α,* and *βTUB* sequences of LN-3 (GenBank accession numbers: MT239573, MT276174, and MT276178) and LN-6 (GenBank accession numbers: MT239574, MT276175, and MT276179) had a 100% identity with *F. acuminatum* (GenBank accession numbers: KR051403, MG826892, and KX880329, and KR047059, JX397865, and MH341246), respectively. A multilocus phylogenetic tree of *F. acuminatum* was constructed, and the results revealed that the grouping of strains LN-3 and LN-6 were supported by a 97% bootstrap value (Figure 5b). The *ITS*, *TEF-1α,* and *Fu* sequences of LN-27 (GenBank accession numbers: MT921794 and MW517798) had a 99%, 87%, and 90% identity with *F. sporotrichioides* (GenBank accession numbers: MT921794, MH582265, and MT921794), respectively. A multilocus phylogenetic tree of *F. sporotrichioides* was constructed, and the results indicated that the grouping of strain LN-27 was supported by a 96% bootstrap value (Figure 5c). Furthermore, the *ITS* and *LSU* sequences of QY3-1 (GenBank accession numbers: MZ571930 and MZ572963) and QY9-1 (GenBank accession numbers: MZ571931 and MZ572964) shared a 98.76%, 100%, and 100% identity with *F. tricinctum* (GeneBank accession numbers: MN856343.1, KT224255.1, and MH868113.1), respectively. A multilocus phylogenetic tree of *F. tricinctum* was constructed, and the results revealed that the grouping of strains QY3-1 and QY9-1 was supported by a 100% bootstrap value (Figure 5d).

## 4. Discussion

A variety of fungal diseases affect the walnut tree, severely reducing walnut output. Previous research found that walnut branch blight is caused by a variety of pathogens, the most common of which was Botryosphaeriaceae, *Cytospora*, and *Diaporthe*. Botryosphaeriaceae fungi have been identified in major walnut-producing countries such as Southern Spain [6,8], the United States [8,10], China [9], Iran [31], Turkey [32], Chile [33], and the Czech Republic [34]. This research, on the other hand, describes *Fusarium* pathogens of walnut branch blight in Longnan, China. Many *Fusarium* species are well-known plant pathogens that may also operate as secondary invaders, and many of these species produced mycotoxins that can contaminate cereal grains [35]. Similarly, *Fusarium* species, as well as members of newly segregated and closely related genera such as *Bisifusarium* and *Neocosmospora* [36], are recognised pathogens of deadly animal and human diseases [18,29]. The morphological flexibility of *Fusarium* species is widely documented. Thus, for convincing species identification, consistent culture media and methods, as well as multi-locus molecular data, are now required [37]. The shape and development of chlamydospores, the presence or absence of sporodochia, and the type of macro- and microconidia are all relevant morphological characteristics [17]. *Fusarium* species have traditionally been recognized using morphological characteristics and pathogenicity tests [17]. Many studies have also shown that the morphological characteristics of *Fusarium* strains are rarely stable within a species [17]. DNA sequence analysis for species identification has gained popularity since early 2000 [38]. The most commonly used sequences to distinguish *Fusarium* spp. are portions of genomic sequences encoding translation elongation factor-1α (*TEF-1α*) [39], β-tubulin (*βTUB*) [40], calmodulin (*CAL*) [24], the intergenic spacer region (*IGS*) [41], DNA-directed RNA polymerase II largest (RPB1), and second-largest subunit (RPB2) [20,38]. PCR can be used to identify *Fusarium* species, either as an alternative or addition to morphological identification. It has been shown to be effective for identifying *Fusarium* species [42,43,44]. Species–specific primers have been designed and used for PCR detection and the screening of *F. graminearum* [45], *F. pseudograminearum* [46], *F. acuminatum* [47], *F. avenaceum* [45], *F. crookwellense* [48], *F. equiseti* [49], *F. poae* [50], and *F. tricinctum* [51]. Despite the fact that some writers suggest that PCR may be used for the routine detection and identification of *Fusarium* species without the necessity for isolation and morphological analysis of this fungus [52], others disagree. This study compared and analyzed the sequences in GenBank, reconstructed the phylogenetic tree, defined the species name from the perspective of molecular phylogeny according to the branch unit where the strain is located, and used dual methods to identify walnut branch blight, ensuring accuracy, convenience, and reliability. Combining the results of morphological and molecular evidence, it is possible to infer that strains LN-1 and LN-19 are *F. avenaceum*, strains LN-3 and LN-6 are *F. acuminatum*, strain LN-27 is *F. sporotrichioides,* and strains QY3-1 and QY9-1 are *F. tricinctum*.

In general, the environment is important in plant pathogen infection [53]. Most studies agree that disease epidemics are influenced by climate, notably, rainfall, humidity, and dew; nevertheless, constant high-humidity conditions might favour the rapid development and spread of walnut branch blight [54]. This life cycle should be used to determine the critical moments with respect to the infection of the pathogens to carry out effective management strategies against the disease. The results of two pathogenicity tests showed that the higher severity of branch blight in the detached leaf assay compared to in vivo clearly indicated that the high humidity and other environmental conditions promoting microbial growth under controlled conditions enhanced blight infection [53]. In addition, our experiment discovered that the symptoms of disease were similar to those of walnut branches under natural conditions, but pathogens were more likely to infect the young shoots of walnuts through wounds than the perennial mature branches, compared to the symptom of the onset and the adult stage of the shoots. Because of walnut trees’ long juvenility period and the complexity of environmental conditions [55], there are differences in the symptoms of walnut branches under natural conditions, which may be related to the differences in the degree of lignification of the branches, the thickness of the cortex, and the growth potential, and more research is needed to determine this. *Fusarium* is mostly identified using morphological methods; however, its culture characteristics are readily influenced by environmental changes, making morphological identification challenging. By understanding the diversity of *Fusarium* species associated with the disease, we will be able to pursue studies on biology, epidemiology, and control to establish optimum management strategies to prevent infections. The occurrence of walnut branch blight has reduced the yield and quality of walnut, which has affected the planting area in Longnan. This study provides a theoretical basis for future research into the prevention of the branch blight disease of walnut. To further understand the epidemiology of the branch blight disease, more knowledge on *Fusarium* spp. biology and interactions with hosts is required. These findings will aid in the diagnosis of walnut branch blight as well as the development of effective management measures in response to the advent of novel diseases.

## 5. Conclusions

In this study, the fungus *Fusarium* of a shoot blight disease of walnut was investigated in China. Because the incidence of walnut disease in this area has increased in recent years, the aim of this study was to isolate and identify the causal pathogens of walnut branch blight in China using morphology- and molecular-based methods to provide a consistent diagnosis. *F. avenaceum* was previously identified as the causative agent of the brown apical necrosis disease of walnut in Hubei, China [56]. This is the first time that *F. acuminatum*-, *F. sporotrichioides*-, and *F. tricinctum*-caused walnut branch blight has been reported in China.

## Figures and Tables

**Table 1:** The sizes of conidia and chlamydospores of *Fusarium*.

*Fusarium* Species		*F. avenaceum*		*F.acuminatum*		*F. sporotrichioides*	*F. tricinctum*	
Isolates		LN-1	LN-19	LN-3	LN-6	LN-27	QY3-1	QY9-1
Macroconidia	Size ^a^ (mm)	33.3 ~ 83.2 × 7.3 ~ 12.7	32.6 ~ 67.0 × 7.3 ~ 15.0	33.5 ~ 54.0 × 8.8 ~ 13.7	40.2 ~ 61.1 × 8.2 ~ 13.7	14.9 ~ 28.7 × 3.2 ~ 8.8	20.1 ~ 37.6 × 2.9 ~ 6.4	20.2 ~ 31.1 × 3.4 ~ 7.0
*n* = 50	Mean ^b^ (mm)	48.7 (± 14.3 ^c^) ~ 10.6 (±1.4)	54.2 (±8.5) ~ 10.4 (±1.7)	42.4 (±6.5) ~ 11.4 (±1.5)	47.5 (±5.8) ~ 11.1 (±1.3)	22.1 (±3.7) ~ 5.2 (±1.0)	27.1 (± 4.1) ~ 4.7 (±0.7)	25.5 (±2.9) ~ 5.0 (±0.7)
Microconidia	Size (mm)	7.4 ~ 15.6 × 2.3 ~ 3.6	7.3 ~ 14.6 × 2.0 ~ 4.3	6.9 ~ 15.5 × 2.2 ~ 4.9	7.0 ~ 13.7 × 1.5 ~ 4.3	6.3 ~ 14.1 × 1.7 ~ 4.9	5.9 ~ 18.0 × 1.7 ~ 4.9	10.1 ~ 20.5 × 2.1 ~ 6.8
*n* = 50	Mean (mm)	9.9 (±2.1) ~ 2.8 (±0.3)	11.3 (±1.9) ~ 3.0 (±0.6)	10.0 (±1.8) ~ 2.9 (±0.6)	11.0 (±1.8) ~ 3.5 (±0.5)	9.7 (±1.8) ~ 3.0 (±0.7)	11.9 (±3.0) ~ 3.0 (±0.5)	13.8 (±2.5) ~ 3.5 (±0.6)
Chlamydospores	Size (mm)	7.3 ~ 17.6 × 6.0 ~ 14.0	12.1 ~ 20.4 × 7.4 ~ 15.7	7.0 ~ 17.3 × 5.3 ~ 15.9	9.0 ~ 20.3 × 5.7 ~ 14.8	7.0 ~ 16.2 × 5.0 ~ 14.5	11.9 ~ 27.6 × 8.5 ~ 19.0	10.6 ~ 24.4 × 7.3 ~ 18.2
*n* = 50	Mean (mm)	8.9 (±2.2) ~ 12.7 (±1.9)	10.9 (±1.9) ~ 15.9 (±1.9)	9.1 (±2.4) ~ 11.8 (±2.2)	8.9 (±2.6) ~ 11.9 (±2.4)	9.9 (±2.6) ~ 7.3 (±2.3)	17.7 (±3.7) ~ 12.5 (±2.7)	16.7 (±3.1) ~ 12.9 (±2.4)

^a^ Size is the size range of the spore dimensions; ^b^ mean is the average of the spore dimensions; ^c^ the values are mean ± standard deviation.

**Table 2:** *Fusarium* species used in the multilocus phylogenetic analysis and their GenBank accession numbers.

*Fusarium* Species	Isolates	GenBank Accession Number ^a^				
		*ITS*	*TEF-1α*	*βTUB*	*Fu*	*LSU*
*F. avenaceum*	LN-1	MT239572	MT276173	MT276177	\	\
	LN-19	MT239575	MT276176	MT276180	\	\
*F.acuminatum*	LN-3	MT239573	MT276174	MT276178	\	\
	LN-6	MT239574	MT276175	MT276179	\	\
*F. sporotrichioides*	LN-27	MT921794	MW517798	\	MT921794	\
*F. tricinctum*	QY3-1	MZ571930	\	\	\	MZ572963
	QY9-1	MZ571931	\	\	\	MZ572964

^a^*ITS*, internal transcribed spacer region; *TEF-1α*, translation elongation factor 1-α; *βTUB*, β-tubulin; *Fu*, a pair of genus-specific primer Fu3/Fu4 for *Fusarium* was designed, based on 18S rDNA and internal transcribed spacer ITS2 sequence; *LSU*, large subunit rDNA.

## Data Availability

Not applicable.

## Supplementary Materials

The following supporting information can be downloaded at: https://www.mdpi.com/article/10.3390/pathogens12070970/s1, Figure S1: Cultural characteristic of Fusarium spp. reisolated (left, upper view; right, dorsal view). a: LN-1. b: LN-19. c: LN-3. d: LN-6. e: LN-27, f: QY3-1, g: QY9-1.
