# Supplementary material for: Molecular Identification and Characterization of Fusarium Associated with Walnut Branch Blight Disease in China
Source: Pathogens. 2023 Jul 24;12(7):970. doi: 10.3390/pathogens12070970 (PMC10384706; doi:10.3390/pathogens12070970)
Supplement: Supplementary file 1 [file pathogens-12-00970-s001.zip › pathogens-2485885-supplementary.pdf]

pathogens

Article

Molecular Identification and Characterization of *Fusarium* Associated with Walnut Branch Blight Disease in China

Ting Ma, Chengde Yang *, Fengfeng Cai and Richard Osei

Biocontrol Engineering Laboratory of Crop Diseases and Pests, College of Plant Protection, Gansu Agricultural University, Lanzhou 730070, China; mating251525@gmail.com (T.M.); 18394280709@163.com (F.C.); nanasei2000@gmail.com (R.O.)
* Correspondence: yangcd@gsau.edu.cn

Supplementary material

The data about identification of strains reisolated

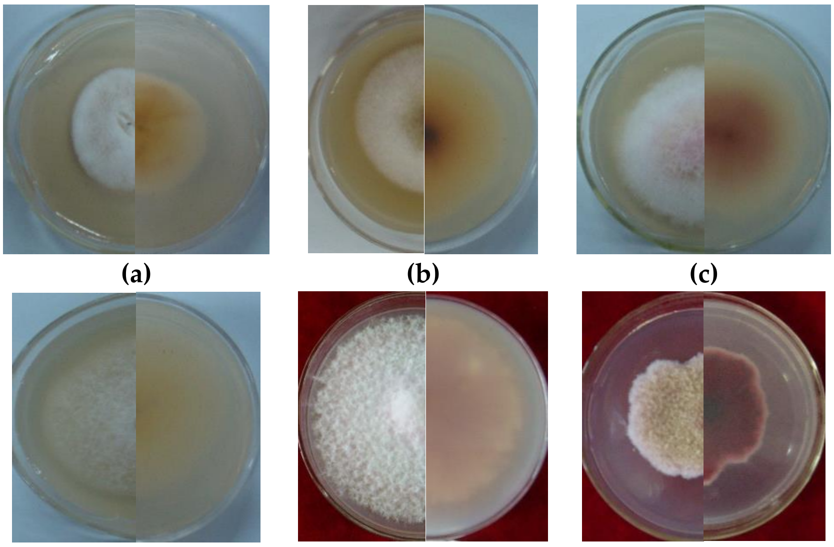

(a) (b) (c) (d) (e) (f)

Citation: Ma, T.; Yang, C.; Cai, F.; Osei, R. Molecular Identification and Characterization of *Fusarium* Associated with Walnut Branch Blight Disease in China. *Pathogens* **2023**, *12*, x. https://doi.org/10.3390/xxxxx

Academic Editor(s): László Kredics

Received: 19 June 2023
Revised: 12 July 2023
Accepted: 12 July 2023
Published: date

Copyright: © 2023 by the authors. Submitted for possible open access publication under the terms and conditions of the Creative Commons Attribution (CC BY) license (https://creativecommons.org/licenses/by/4.0/).

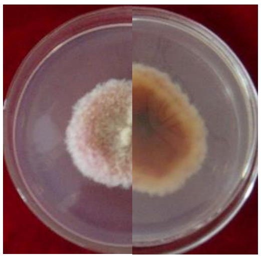

(g)

Figure S1. Cultural characteristic of *Fusarium* spp. reisolated (left, upper view; right, dorsal view). a: LN-1. b: LN-19. c: LN-3. d: LN-6. e: LN-27, f: QY3-1, g: QY9-1.
